# Draft Genome Sequence of Weissella viridescens UCO-SMC3, Isolated from the Slime of *Helix aspersa* Müller Snails

**DOI:** 10.1128/MRA.01654-18

**Published:** 2019-03-14

**Authors:** Apolinaria Garcia-Cancino, Leonardo Albarracin, Marcela Espinoza-Monje, Jorge Campos-Martin, Valeria Garcia-Castillo, Yuka Nakano, Wakako Ikeda-Ohtsubo, Cristian Guitierrez-Zamorano, Hidetoshi Morita, Haruki Kitazawa, Julio Villena

**Affiliations:** a Laboratory of Bacterial Pathogenicity, Faculty of Biological Sciences, University of Concepcion, Concepcion, Bio Bio, Chile; b Reference Centre for Lactobacilli (CERELA-CONICET), Tucuman, Argentina; c Scientific Computing Laboratory, Computer Science Department, Faculty of Exact Sciences and Technology, National University of Tucuman, Tucuman, Argentina; d Food and Feed Immunology Group, Laboratory of Animal Products Chemistry, Graduate School of Agricultural Science, Tohoku University, Sendai, Japan; e Livestock Immunology Unit, International Education and Research Centre for Food Agricultural Immunology (CFAI), Graduate School of Agricultural Science, Tohoku University, Sendai, Japan; f Graduate School of Environmental and Life Science, Okayama University, Okayama, Japan; Queens College

## Abstract

This report describes the draft genome sequence of Weissella viridescens UCO-SMC3, isolated from Helix aspersa Müller slime. The reads were generated by a whole-genome sequencing (WGS) strategy on an Illumina MiSeq sequencer and were assembled into contigs with a total estimated size of 1,612,814 bp. A total of 2,455 genes were predicted, including 2,301 protein-coding sequences. The draft genome sequence of W. viridescens UCO-SMC3 will be useful for further studies of specific genetic features and for understanding the mechanisms of its beneficial properties in the skin.

## Abstract (precis)

This report describes the draft genome sequence of Weissella viridescens UCO-SMC3, isolated from Helix aspersa Müller slime. The reads were generated by a whole-genome sequencing (WGS) strategy on an Illumina MiSeq sequencer and were assembled into contigs with a total estimated size of 1,612,814 bp.

## ANNOUNCEMENT

In recent years, skin care products infused with snail slime have become popular. The efficacy of snail secretion in wound healing has been proven both *in vitro* and by clinical studies (1, 2). It was reported that snail mucus components are able to stimulate collagen and elastin formation in the skin and to improve the protection against photoaging and free radical-mediated damage (3, 4).

We hypothesized that local microbiota can be involved in the beneficial effects of snail slime. Therefore, with the aim of improving our understanding of the factors involved in the skin protection achieved by snail slime, we isolated lactic acid bacteria (LAB) from the slime of the garden snail Helix aspersa Müller. Adult snails were fasted for 12 h and then stimulated for mucosal secretion in a biosafety cabinet. Mucous secretions were collected and seeded in de Man-Rogosa-Sharpe (MRS) agar for the isolation of LAB at 37°C and 10% CO_2_ for 48 h. Among the LAB isolated from snail slime, the UCO-SMC3 strain was selected because of its ability to inhibit the growth of the skin-associated pathogens Cutibacterium acnes and Staphylococcus aureus *in vitro*. The 16S RNA sequence analysis indicated that the UCO-SMC3 strain belongs to the species Weissella viridescens.

A single colony of strain UCO-SMC3 was picked for culturing prior to DNA isolation. W. viridescens UCO-SMC3 was cultured for 12 h at 37°C (final log phase) in MRS broth (Oxoid, Cambridge, UK), and genomic DNA isolation was performed as described by Azcárate-Peril and Raya (5). The genomic DNA of W. viridescens UCO-SMC3 was sequenced with the 2 × 150-bp paired-end read length sequencing protocol of the Illumina MiSeq platform. The generated sequencing reads were filtered to remove low-quality reads and were then assembled with A5-miseq (6). The sequencing protocol generated approximately 74.0× coverage of the genome. Strain UCO-SMC3 contained 19 contigs with a G+C content of 41.4% and a total length of 1,612,814 bp. The Rapid Annotations using Subsystems Technology (RAST) server was used for functional annotation of predicted genes (7). A total of 2,455 genes were predicted, including 2,301 protein-coding sequences, 60 tRNAs, 19 rRNAs, and 3 noncoding RNAs (ncRNAs). In addition, 1 clustered regularly interspaced short palindromic repeat (CRISPR) array was annotated in the genome, which was confirmed by using CRISPRFinder (8). Default parameters were used for bioinformatic analysis.

RAST analysis revealed that the W. viridescens UCO-SMC3 genome has genes for resistance to trimethoprim and fluoroquinolones. The genome was further analyzed with BAGEL4 for the detection of bacteriocins (9), but no bacteriocin genes were detected.

Two genes of collagen adhesins (*cna1* and *cna2*) and an extracellular matrix-binding protein gene (*ebh*) that promotes bacterial attachment to both soluble and immobilized forms of fibronectin were found in W. viridescens UCO-SMC3. The *ssp5* gene for the agglutinin receptor that is able to bind sialic acid residues of salivary agglutinin in a calcium-dependent manner was also found in the UCO-SMC3 genome. In addition, clusters of genes involved in the biosynthesis of tetrahydrofolate, riboflavin, and biotin were found in strain UCO-SMC3.

The draft genome sequence of W. viridescens UCO-SMC3 will be useful for further studies of specific genetic features and for understanding the mechanisms of its beneficial properties in the skin.

### Data availability.

This whole-genome shotgun project has been deposited at DDBJ/ENA/GenBank under the accession number RHGY00000000. The version described in this paper is version RHGY01000000. The SRA/DRA/ERA accession number is ERP111538. The BioSample and BioProject numbers are SAMN09991636 and PRJNA489880, respectively.
